# Prevalence of Aphid-Transmitted Potyviruses in Pumpkin and Winter Squash in Georgia, USA

**DOI:** 10.3390/v17020233

**Published:** 2025-02-08

**Authors:** Nirmala Acharya, Manish Kumar, Sudeep Bag, David G. Riley, Juan C. Diaz-Perez, Alvin M. Simmons, Timothy Coolong, Theodore McAvoy

**Affiliations:** 1 Department of Horticulture, University of Georgia, Tifton, GA 31793, USA; 2 Department of Plant Pathology, University of Georgia, Tifton, GA 31793, USA; 3 Department of Entomology, University of Georgia, Tifton, GA 31793, USA; 4 U.S. Vegetable Laboratory, Agricultural Research Service, United States Department of Agriculture, Charleston, SC 29414, USA; 5 Department of Horticulture, University of Georgia, Athens, GA 30602, USA

**Keywords:** cucurbit, aphids, high-throughput sequencing, zucchini yellow mosaic virus, papaya ringspot virus, prevalence, pumpkin, winter squash

## Abstract

Viruses are a major pathogen challenging the sustainable production of cucurbits worldwide. Pumpkin and winter squash showed severe virus-like symptoms during the fall of 2022 and 2023 in Georgia, USA. Symptomatic leaves were collected from the field and processed for small RNA sequencing for virus identification using high-throughput sequencing (HTS). HTS analysis revealed the presence of two aphid-transmitted viruses (ATVs), zucchini yellow mosaic virus (ZYMV) and papaya ringspot virus (PRSV), along with three whitefly-transmitted viruses, cucurbit chlorotic yellows virus, cucurbit yellow stunting disorder virus, and cucurbit leaf crumple virus. The results of our study suggest a significant shift in ATV’s abundance in these two crops between 2022 and 2023. According to the qPCR data in the fall of 2022, pumpkins experience an incidence of 56.25% and 31.25% of PRSV and ZYMV, respectively. Similarly, winter squash shows an incidence of 50% and 32.14% of PRSV and ZYMV, respectively. Mixed infection of both viruses was also observed in these two crops. In 2023, we observed a predominance of ZYMV in pumpkin and winter squash (61.25% and 42.50%, respectively). However, PRSV was not detected in pumpkins, and it was detected at a negligible level (0.62%) in winter squash using qPCR. Phylogenetic analysis of ZYMV-encoded coat protein (CP) and helper component-protease (HC-Pro) from Georgia suggests a close relationship with the European isolates. Conversely, PRSV-encoded CP and NIa-VPg show a more diverse evolutionary history. Overall, this research will provide valuable insights into the dynamics of ZYMV and PRSV in pumpkin and winter squash crops within the southeastern United States.

## 1. Introduction

Cucurbits are an important family of vegetable crops worldwide. Global cucurbit vegetable production that includes cucumbers (*Cucumis sativus* L.), gherkins (*Cucumis anguria* L.), pumpkins (*Cucurbita maxima* Duchesne), squash (*Cucurbita pepo* L.), and gourds (*Lagenaria siceraria* (Molina) Standl.) is estimated to be 117.53 million metric tons in the year 2023, accounting for 13.8% of all the vegetables produced globally [1]. China and India are leading producers of cucurbits, followed by Ukraine, Russia, the United States of America (USA), and Spain. In 2023, the United States produced 4.5 million metric tons of cucurbits, including above-mentioned cucurbits as well as cantaloupe (*Cucumis melo* L.) and honeydew (*Cucumis melo* L.), watermelon (*Citrullus lanatus* Thunb. (Matsum.) and Nakai), grown in a total area of 141,600 ha accounting for an estimated economic value of USD 968.5 million [2]. This highlights the economic importance of cucurbits globally and in the USA. Summer squash and cucumber fruits are often harvested at immature stages of development, while pumpkin and winter squash fruits are typically harvested at their physiological maturity, allowing for long-term storage. Pumpkin and winter squash seeds are snack food and ground to make sauces, and the flowers are fried and eaten directly or incorporated as a seasoning for food [3,4]. In the USA, pumpkin and winter squash also have cultural significance, being used for edible consumption and decorations, for the Halloween and Thanksgiving holidays [5]. Across the USA, pumpkin and winter squash commercial production is concentrated in central and northern states but not in southern states due to several biotic and abiotic factors, with virus diseases being one of the key biotic threats [6,7].

**Figure 1:**
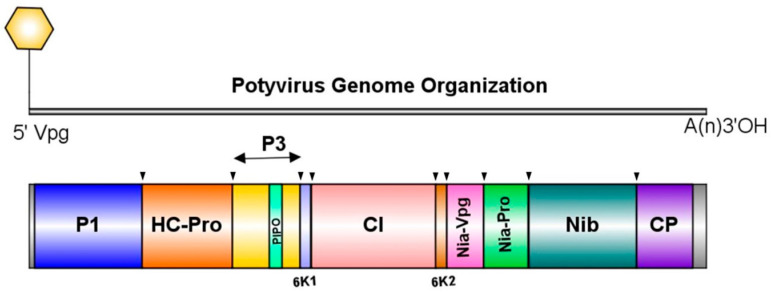
Genome organization of potyviruses; inverted triangular arrows indicate polyprotein cleavage sites. The P1 is a serine protease, an accessory factor for viral amplification. The helper-component proteinase (HC-Pro) is a cysteine protease that has a role in viral transmission, RNA silencing suppression, and systemic movement. The P3 and 6K2 are involved in viral replication and multiplication. The CI has ATPase and helicase activity; it has a role in particle disassembly and virus movement. The 6K2 anchors the replication apparatus to ER-like membranes and induces the formation of viral replication vesicles. The NIa-Vpg has a role in viral replication, RNA translation, and movement. The NIa-pro is a trypsin-like serine protease and has a role in the proteolytic cleavage of the potyviral polyprotein. It also functions in host specificity, DNase activity, RNA binding, replication, and multiplication. The NIb is RNA-dependent RNA polymerase and has a role in viral replication and multiplication. The coat protein (CP) encapsulates the virus’s RNA genome. In association with HC-Pro, CP has a role in seed transmission, cell–cell and systemic movement, virus assembly, and host adaptation. Pretty Interesting *Potyviridae* ORF (PIPO) has a primary role in viral movement. These virus-encoded proteins have a role in the movement, pathogenicity, symptom development, and resistance breakdown [16].

There are more than 90 different viruses infecting cucurbits across the world [8,9,10,11,12,13], and the majority of them are transmitted by aphids and whiteflies [14]; aphid-transmitted viruses (ATVs) from several families, including Potyviridae, the largest plant-infecting RNA virus family [15]. Potyviruses have a positive sense single-stranded RNA (ssRNA) genome with about 10 kb length, including 5′ and 3′ non-translated regions. The genome has one open reading frame (ORF) encoding a single polyprotein precursor that is subsequently processed by three virally encoded proteases to produce 10 functional small mature proteins [16]. The genome organization of potyviruses is shown in Figure 1.

In the USA, more than 25 viruses have been reported to infect cucurbits [17,18,19]. Field surveys in Oklahoma, USA, found a high incidence of three potyviruses, papaya ringspot virus (PRSV), watermelon mosaic virus-2 (WMV-2), and zucchini yellow mosaic virus (ZYMV) in cucurbits during 2008–2010 [20]. When cucurbit leaf samples from 10 different states in the southern USA were tested, 13 viruses were detected between 2010 and 2011; among them, the same three potyviruses were frequently presented [17]. These three viruses and cucumber mosaic virus (CMV) are also reported in Georgia’s list of widely prevalent viruses [21]. Over recent years, whitefly-transmitted viruses (WTVs), including Begomoviruses, Criniviruses, and Ipomoviruses, have become endemic in Georgia, USA [18]. In Georgia, USA, the cucurbit-infecting common WTVs are cucurbit chlorotic yellow virus (CCYV), cucurbit yellow stunting disorder virus (CYSDV) [Criniviruses], and cucurbit leaf crumple virus (CuLCrV) [Begomovirus]. Additionally, there are reports of several novel viruses emerging in the region [22,23,24,25,26]. For the last few years, cucurbit-infecting virus research conducted in Georgia, USA, has predominantly focused on WTVs. In contrast, studies examining the prevalence of ATVs in cucurbits have been lacking for over a decade. Moreover, most of the existing studies have centered on summer squash, leaving a significant knowledge gap regarding the prevalence of ATVs in pumpkin and winter squash. This study investigates the occurrence, prevalence, and emergence of ATVs in pumpkin and winter squash crops grown in Georgia, USA. Our study will contribute as an important step toward the understanding complex interactions between aphid- and whitefly-transmitted viruses in cucurbits crops.

## 2. Materials and Methods

### 2.1. Crop Production

Commercial cultivars of pumpkin and winter squash seeds were sown directly in the field in the second week of August at the coastal plain experiment station, University of Georgia, Tifton, Georgia (latitude 31°29′14″ N and longitude 83°31′10″ W) in the fall of 2022 and 2023. The cultivars of pumpkin and winter squash grown in the experimental field are shown in Table S1. The crop management practices were followed as recommended by the UGA Extension Services (https://extension.uga.edu/publications/detail.html?number=B1180) (accessed on 7 January 2025) (l) (Table S2). Seeds were sown using a double-row system, with five plants per row and ten plants per plot.

### 2.2. Symptoms Observation

In both years, cucurbit plants were observed visually for the development of virus symptoms and assessed weekly. At 45 days after seed sowing (DAS), infected plants showing severe symptoms, including leaf yellowing, chlorotic spots, shoestring, curling, crumpling, mosaic, adaxial, and abaxial blistering on leaves, were collected.

### 2.3. Sample Collection

In fall 2022, pumpkin (n = 32) and winter squash (n = 56) symptomatic leaves were collected at 45 DAS. In 2023, symptomatic leaves of pumpkin (n = 80) and winter squash (n = 160) were collected at 45 DAS. The third and seventh leaves from the apical terminal leaf of every alternate plant from each plot were collected, and the upper and lower leaves for each plant were combined together. Furthermore, pumpkin (n = 4) and winter squash (n = 2) leaves with distinct virus symptoms in the field were collected for use in the small RNA (sRNA) analysis. All the samples were surface cleaned with distilled water to remove dirt, and 100 mg of each leaf sample was stored in a −80 °C deep freezer until further processing.

### 2.4. RNA Extraction and High-Throughput Sequencing (HTS) of sRNA

Total RNA was extracted from leaves’ tissues using the total RNA extraction kit (Spectrum, Millipore Sigma, St. Louis, MO, USA) following the manufacturer’s instructions. The concentration and purity of isolated RNA were assessed using a NanoDrop Spectrophotometer (Thermo Fisher Scientific, Waltham, MA, USA). Extracted total RNA with a concentration > 400 ng/μL was sent to Novogene (Sacramento, CA, USA) on dry ice for sRNA analysis using HTS. sRNA libraries were constructed using an Illumina sRNA sequencing platform Novogene, with single-end reads of 1 × 50 bp [27].

### 2.5. In Silico Data Analysis

sRNAs generated through HTS were analyzed to identify the viruses using CLC Genomics Workbench v23.0.2. (Qiagen, Redwood City, CA, USA). The adapter sequences, low-quality sequences, and sequences with more than two ambiguous nucleotides were excluded. Reads ranging from 18 to 30 nucleotides in length were selected. The contigs length of a minimum of 50 nucleotides was assembled for de novo analysis. De novo assembly was carried out with established guidelines such as mapping to create a simple contig sequence (fast), mismatch cost-2, insertion cost-3, deletion cost-3, alignment mode-local, and minimum contig length-50. A local virus database was compiled using the Create Database feature of CLC Genomics Workbench by retrieving relevant sequences from the National Center for Biotechnology Information (NCBI) (https://www.ncbi.nlm.nih.gov/). Contigs were compared to all database sequences for a possible match using BLASTn with default settings. Contigs aligning with non-plant virus sequences were excluded from further analysis [24].

### 2.6. Construction of Viral Genome Sequence and Coverage Maps

In CLC Genomics Workbench, reference-based mapping was used to assemble consensus sequences with the following parameters: mismatch cost (2), insertion cost (3), and deletion cost (3). sRNA reads were aligned with the reference sequences of ATV’s potentially present in the samples. Graphical visualizations of genome assembly coverage were created, displaying read tracks that represent the maximum, minimum, and average coverage values for each region. The alignment of consensus sequences with the reference genome was checked for discrepancies with the CLC Genomics Workbench.

### 2.7. Molecular Validation of HTS Results

Total nucleic acid (TNA) from the 100 mg of leaf samples was extracted using magnetic bead technology using tissue Bead Mill 24 Homogenizer (Thermo Fisher Scientific) at a speed of 3.55 m/s for 45 s to ensure high-quality nucleic acids for downstream applications. Leaf samples were mechanically disrupted in 2 mL screw-cap tubes with 200 μL 4 M guanidine thiocyanate (GTC) buffer (pH 5.0) supplemented with two ceramic beads of 2.8 mm in size (Omni International, Kennesaw, GA, USA). Subsequently, the homogenate was centrifuged at 10,000× *g* for 30 s. The supernatant (115 μL) was mixed with RNA-binding magnetic beads and processed using the MagMAX 96 viral RNA kit using the KingFisher Flex Purification System (Thermo Fisher Scientific). Furthermore, TNA was eluted with 100 μL of nuclease-free water [26]. The complementary DNA (cDNA) was synthesized using the iScript cDNA Synthesis Kit (Bio-Rad, Hercules, CA, USA) using virus-specific primers (Table 1) followed by the quantitative polymerase chain reaction (qPCR) assay to detect the presence of targeted viruses in the sample.

Briefly, for cDNA synthesis, 4 μL of 5X iScript reaction buffer mix and 1 μL of iScript reverse transcriptase (RT) enzyme were mixed with 100 ng/μL TNA as a template, and the final volume of 20 μL was adjusted using nuclease-free water per reaction (Bio-Rad). The reaction mixture was denatured at 25 °C for 5 min and incubated at 45 °C for 20 min, followed by RT inactivation at 95 °C for 1 min in a T100 thermal cycler (Bio-Rad). qPCR was performed using the master mix, consisting of 10 μL of SYBR green mix (Bio-Rad), 0.5 μM per μL of each forward and reverse primer, and 6 μL of nuclease-free water resulting in 18 μL for each sample. In a 96-well plate, 18 μL master mix was added to each respective well, followed by 2 μL of cDNA. For PRSV detection, the qPCR was set at the prior established profile with slight modifications [16]: 95 °C for 3 min (initial denaturation), followed by 95 °C 10 s for (denaturation), annealing temperature at 58 °C for 30 s, 65 °C for 5 s (initial extension), and 95 °C for 30 s (final extension); similarly, for ZYMV detection, 95 °C for 5 min (initial denaturation), followed by 95 °C 15 s (denaturation), and primer-specific annealing temperature at 60 °C for 60 s, 65 °C for 5 s (initial extension), and 95 °C for 30 s (final extension) [28] in the C1000 touch thermal cycler CFX96TM real-time system (Bio-Rad). The melt curve was analyzed at the end of each cycle to determine the specificity of the qPCR assay using CFX Maestro software, v 2.3 (Bio-Rad).

### 2.8. Target Gene Amplification and Sanger Sequencing

Coat protein (CP) and NIa-Vpg ORFs were used to generate PRSV-gene-specific primers to validate the presence of viruses in the samples. Similarly, CP and helper component-protease (HC-Pro) ORF regions were targeted for ZYMV. For PRSV detection, qPCR was set at 94 °C for 2 min (initial denaturation), followed by 94 °C for 45 s (denaturation), and annealing temperature at 58 °C for 45 s, 72 °C for 50 s (initial extension), 72 °C for 5 min (final extension) [16]. For ZYMV, RT-PCR was set at 94 °C for 60 s (initial denaturation), followed by 94 °C for 30 s (denaturation), and an annealing temperature of 58 °C for 30 s, 72 °C for 60 s (initial extension), 72 °C for 5 min (final extension) [28]. For gel electrophoresis, 1% agarose gel was prepared using Tris-Acetate-EDTA (TAE) buffer, and samples were loaded on gel at 60 V for at least an hour. The amplicons were gel-extracted using the QIAquick gel extraction kit (QIAGEN). All the amplicons were of expected size so four PCR products were pooled for pumpkin, and two PCR products were pooled for winter squash for each ORF. The pooled products were then sent to Azenta Life Sciences (South Plainfield, NJ, USA) for bidirectional Sanger sequencing with full-length forward and reverse virus-specific ORF primers. This process was repeated three times to resolve the ambiguous “N” in the sequences.

### 2.9. Phylogenetic Analysis and Nucleotide Sequence Identity

Phylogenetic trees were constructed using 28 full-length ZYMV-encoded CP sequences and 29 HC-Pro sequences. Similarly, 25 full-length CP sequences and 28 NIa-Vpg sequences were used to generate the phylogenetic tree for PRSV (Table S4). Based on nucleotide sequence identity, additional sequences of ZYMV (CP and HC-Pro) and PRSV (CP and NIa-Vpg) were retrieved from the NCBI GenBank. Multiple sequence alignments were performed using the MUSCLE algorithm, followed by phylogeny testing using the 1000 bootstrap method, with the maximum likelihood method using the MEGA-X tool [29]. Sweet potato mild mottle virus (SPMMV) [Genus: Ipomovirus, Z73124] was used as an outgroup for the tree. The final tree was created using uniform rates among the sites, and branches were condensed to 50% with a 1000 bootstrap value. The calculated trees were displayed and analyzed using Tree Explorer implemented in the MEGA-X program. Percent nucleotide identity was calculated, and color-coded heat map matrices were generated using the Sequence Demarcation Tool (SDT) version 1.2 [30].

## 3. Results

### 3.1. Symptomatology

**Figure 2:**
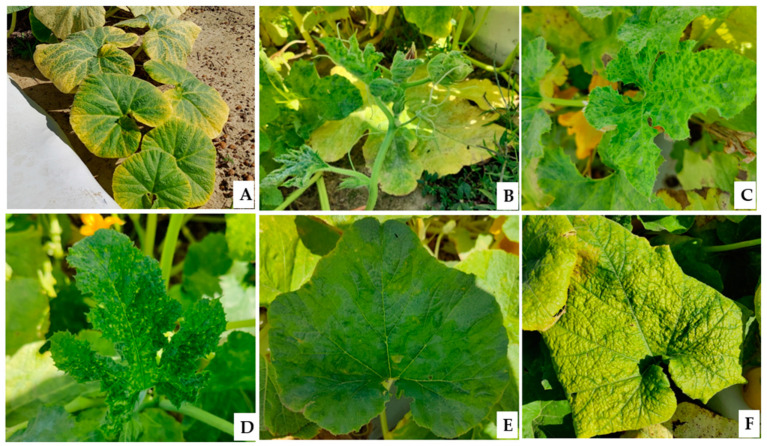
Different symptoms observed on pumpkin and winter squash plants included pumpkin leaf (**A**) marginal yellowing (PK1); (**B**) cupping and shoestring (PK2); (**C**) mosaic and vein clearing (PK3); and (**D**) thickening, curling, crumpling, and vein clearing (PK4). The two symptoms observed in winter squash leaves were (**E**) light and dark green mosaic and blistering (WS1) and (**F**) yellowing, thickening, and inward rolling (WS2).

The pumpkin and winter squash fields observed a wide range of virus-like symptoms. The symptoms on pumpkins were more diverse than those on winter squash. The different types of symptoms observed on pumpkin leaf include (I) marginal yellowing (PK1), (II) cupping and shoestring (PK2), (III) mosaic and vein clearing (PK3), (IV) thickening, curling, crumpling, and vein clearing (PK4) (Figure 2A–D). Similarly, in winter squash, the symptoms observed included (I) light and dark green mosaic and blistering (WS1) and (II) yellowing, thickening, and inward rolling (WS2) of leaf tissues (Figure 2E,F).

### 3.2. Virus Detection Using HTS

HTS generated 20–30 million reads after trimming the adapter and performing quality control. The length distribution of each sample was comparable, with most sRNAs being 21–24 nucleotides (nt) long. Using BLASTn, contigs with a minimum length of 50 base pairs (bp) were compared to virus sequences at the NCBI database. BLASTn search confirmed the presence of two different aphid-transmitted potyviruses, ZYMV and PRSV, in symptomatic samples. The percentage of sRNA sequences by viruses differed among the samples. To define virus presence based on HTS reads, we used a threshold of at least 80% nucleotide identity with the reference genome sequence.

**Figure 3:**
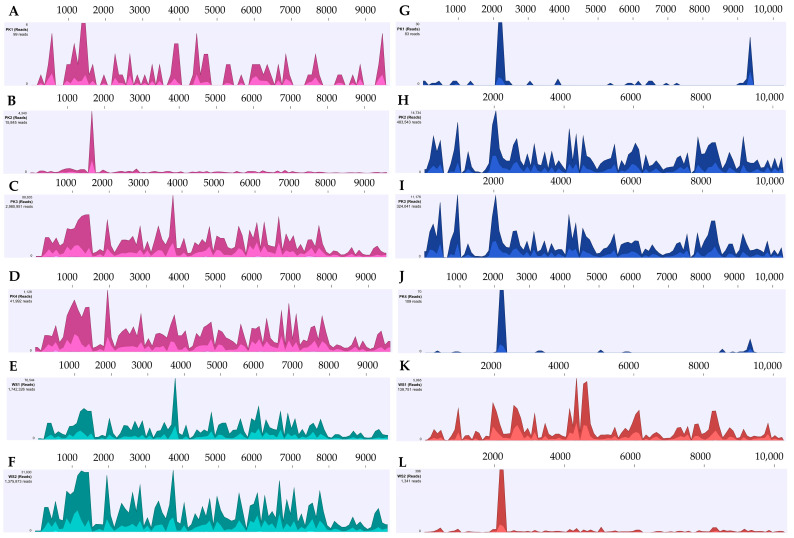
Read coverage maps of the genome of the zucchini yellow mosaic virus (ZYMV) and papaya ringspot virus (PRSV) detected by high-throughput sequencing (HTS) of small RNAs of symptomatic pumpkin and winter squash samples. The data were analyzed using CLC Genomics workbench v23.0.2. The colored heatmap shows the coverage with several reads. A to F represent read coverage maps of the ZYMV for samples with (**A**) marginal yellowing (PK1), (**B**) cupping and shoestring (PK2), (**C**) mosaic and vein clearing (PK3), (**D**) thickening, curling, crumpling, and vein clearing (PK4), (**E**) light and dark green mosaic and blistering (WS1), and (**F**) yellowing, thickening, and inward rolling (WS2). On the other hand, G to L shows read coverage maps of the PRSV for (**G**) PK1, (**H**) PK2, (**I**) PK3, (**J**) PK4, (**K**) WS1, and (**L**) WS2. Genome positions of the virus are presented to scale above the histograms, and the coverage in the number of reads with reference genomes (GenBank accession number NC_003224 for ZYMV and NC_001785 for PRSV) is represented on the Y-axis. The colors in the specified aggregation bucket mean the maximum, average, and minimum coverage values (read counts) from top to bottom.

Near-complete genomes of ZYMV and PRSV were assembled from the sRNA sequences. The nucleotide sequence of ZYMV isolates from pumpkin and winter squash grown in Georgia, USA, shared 89.15–100% and 92.97–93.85%, respectively, identity with reference genome sequence (NC_003224) reported from Taiwan in Luffa cylindrica in BLASTn analysis. Likewise, the nucleotide sequence identity of PRSV isolates from pumpkin and winter squash ranged from 89.72–100% and 82.88–90.10%, respectively, with PRSV reference genome (NC_001785) reported from Taiwan in Cucumis metuliferus (Table 2, Figure 3). In addition, reads from each sample were aligned with the reference sequences of other known viruses that infect cucurbits (Table S3). Three whitefly-transmitted viruses CCYV, CYSDV, and CuLCrV were also detected, which are not in the scope of this paper.

### 3.3. Virus Prevalence and Distribution in Pumpkin and Winter Squash

**Figure 4:**
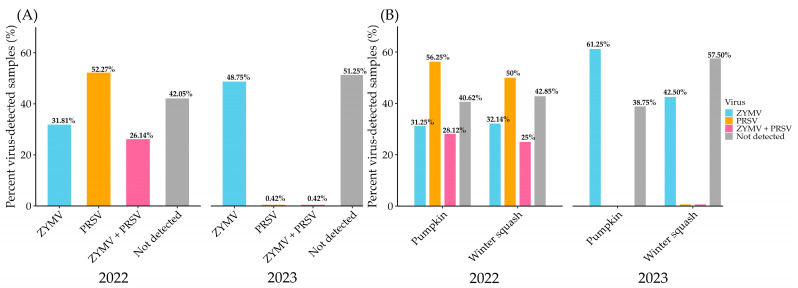
Graphs showing the prevalence of zucchini yellow mosaic virus (ZYMV) and papaya ringspot virus (PRSV): (**A**) bar graphs showing the prevalence of ZYMV and PRSV between the two consecutive years, 2022–2023, among the total samples collected; (**B**) distribution of ZYMV and PRSV in pumpkin and winter squash in 2022–2023.

Overall, in 2022, PRSV (52.27%) was more prevalent than ZYMV (31.81%) in the samples (n = 88) tested. However, 26.14% of plants have mixed infections with both viruses, which were not detected in 42.05% of the samples. In 2023, ZYMV was more prevalent, detected in 48.75% of the total samples (n = 240) analyzed. Notably, PRSV and mixed infection were detected at a negligible level, with only 0.42% of the tested samples. Both viruses were not detected in 51.25% of the total samples tested (Figure 4A). A crop-specific analysis of qPCR data shows the differential incidence of the virus in pumpkin and winter squash. In 2022, in pumpkin samples, ZYMV was present in 31.25%, and PRSV was detected in 56.25% of the tested samples; whereas 28.12% of the samples were mixed infected with both viruses. However, in winter squash, ZYMV was detected in 32.14%, while PRSV was found in 50% of the samples tested. On the other hand, 25% of the samples were detected with mixed infection. The percentage of samples with no detection of either virus was 40.61% and 42.85% for pumpkin and winter squash, respectively. In contemporary, in pumpkin samples collected in 2023, ZYMV was detected in 61.25% of the samples, while no PRSV or mixed infection was detected. In winter squash, ZYMV was detected in 42.50% of samples, and PRSV and mixed infection were detected at an insignificant level (0.62%) of the tested samples. These viruses were not detected in 38.75% of pumpkin and 57.50% of winter squash samples (Figure 4B).

### 3.4. Sequence Analysis and Phylogenetic Tree

The sequence obtained from Sanger’s sequencing was analyzed using NCBI BLASTn (https://blast.ncbi.nlm.nih.gov/). ZYMV-CP sequence is 837 bp (PQ685676 and PQ685677), while HC-Pro is 1368 bp in length (PQ685678 and PQ685679). Similarly, PRSV-CP is 861 bp (PQ685680 and PQ685681) and PRSV-NIa-Vpg is 571 bp in length (PQ685682 and PQ685683). BLASTn results suggested ZYMV-CP (PQ685676 and PQ685677; Georgia) and ZYMV-HC-Pro (PQ685678 and PQ685679; Georgia) showing 99.92% (ON604841; Hungary) and 99.34% (KU244515; Greece) homology with NCBI database sequences. In addition, PRSV-CP (PQ685680 and PQ685681; Georgia) showed 95.33% with MZ099474 (USA), and PRSV-NIa-Vpg (PQ685682 and PQ685683; Georgia) showed 93.65% with KX655867 (Australia) sequence of NCBI GenBank.

**Figure 5:**
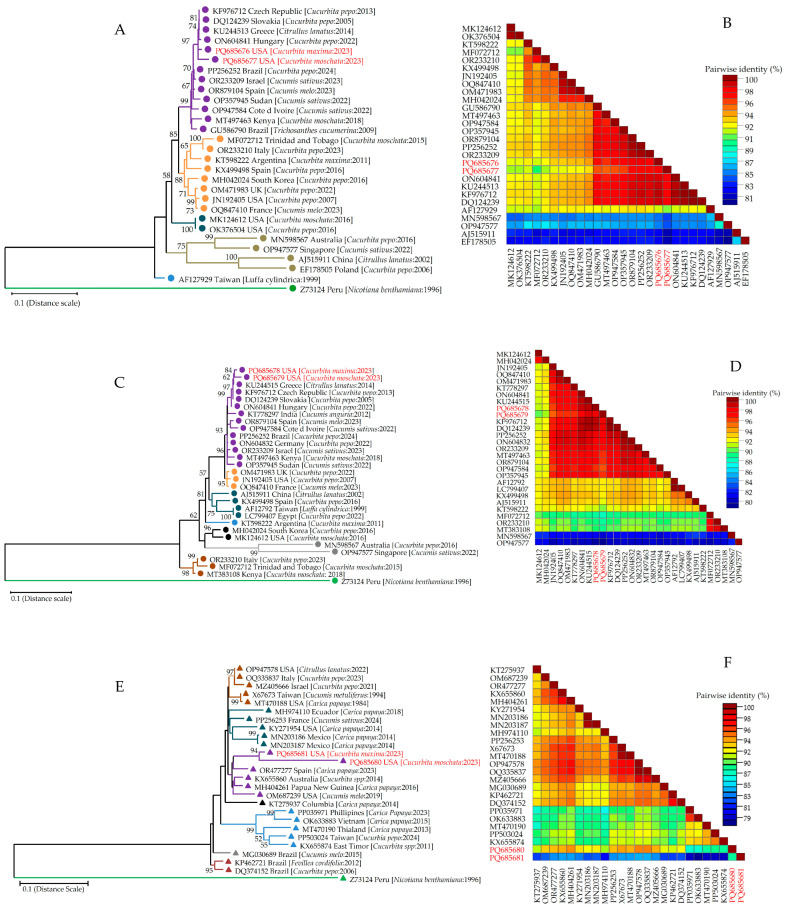

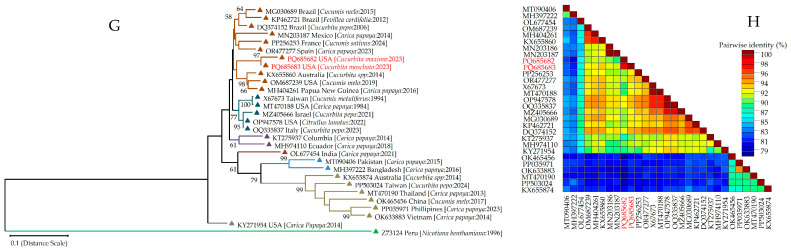
Sequence comparisons of nucleotide sequences of zucchini yellow mosaic virus (ZYMV)-encoded coat protein (CP) and helper component proteinase (HC-Pro) and papaya ringspot virus (PRSV)-encoded CP and NIa-Vpg from pumpkin and winter squash plants in Georgia, USA, with the sequences obtained from the NCBI GenBank. Maximum likelihood (ML) phylogenetic trees deduced in MEGAX using the 1000 bootstrap method. The phylogenetic trees are based on (**A**) 28 sequences of ZYMV-encoded CP, (**C**) 29 sequences of ZYMV-encoded HC-Pro, (**E**) 25 sequences of PRSV CP, and (**G**) 28 sequences of PRSV NIa-Vpg. GenBank accession number, country name, host plant, and year of sample collection are shown on each node. Sweet potato mild mottle virus (SPMMV) [Genus: Ipomovirus, Z73124] was used as an outgroup for the tree. The final tree was created using uniform rates among the sites, and branches were condensed to 50%. The calculated trees were displayed and analyzed using Tree Explorer implemented in the MEGA-X program. Color-coded pairwise identity matrixes generated from (**B**) 28 ZYMV-encoded CP, (**D**) 29 ZYMV-encoded HC-Pro, (**F**) 25 PRSV-encoded CP, and (**H**) 28 PRSV-encoded NIa-Vpg sequences. Each colored cell represents a percentage identity score between two sequences (one indicated horizontally to the left and the other vertically at the bottom).

The phylogenetic dendrogram of ZYMV-CP (PQ685676 and PQ685677; Georgia) identified from pumpkin and winter squash depicted a close relatedness with European isolates (Czech Republic, Greece, Hungary, and Slovakia) (Figure 5A). These results were corroborated with a heat map generated using nucleotide percent identity (Figure 5B). Notably, ZYMV-HC-pro (PQ685678 and PQ685679; Georgia) isolated from pumpkin and winter squash show a similar phylogenetic relationship and percent nucleotide identity with other European isolates (Figure 5C,D). Furthermore, PRSV-CP sequences identified in Georgia, USA, were also checked for generating a phylogenetic dendrogram and heat map. The tree generated here shows a more diverse evolutionary history compared to other ATVs (such as ZYMV). PRSV isolates from Australia, Papua New Guinea Spain, and the USA were closer to PRSV-CP isolated identified in Georgia, USA, suggesting its presence in multiple hosts (Figure 5E,F). Similarly, PRSV-NIa-Vpg-based phylogeny (Figure 5G,H) also shows a similar pattern as depicted in the PRSV-CP-based evolutionary relationship.

## 4. Discussion

Cucurbit production has been significantly impacted by various pathogens, including viruses. Most plant viruses are insect-vectored. In contrast to WTVs, aphid-borne potyviruses such as ZYMV and PRSV are transmitted via non-persistent mode, suggesting aphids might acquire the viruses quicker while feeding on infected plants. Potyviruses are the largest plant-infecting RNA viruses encoding multiple viral proteins. Cucurbit plants infected with ZYMV exhibit symptoms such as leaf yellow mosaic, blistering, shoestring, and fruit distortion. Similarly, PRSV-infected cucurbits show similar symptoms, including leaf filiformity [31]. A mixed infection of ZYMV and PRSV might intensify symptoms severity. Recently, cucurbit-infecting virus research conducted in Georgia has predominantly focused on WTVs. This study presents the dynamics of ZYMV and PRSV in pumpkin and winter squash grown in Georgia, which have been lacking.

In this study, we observed diverse symptoms in pumpkin and winter squash cultivars grown in Georgia, USA, during the fall of 2022 and 2023. Infested plants showed foliar symptoms, including mosaic, mottling, yellowing, chlorotic spots, vein clearing, shoestring, upward curling, crumpling, blistering, and deformation of leaves (Figure 2). In open-field production systems, where plants are exposed to a range of insect vectors, mixed infections are common. In the USA, earlier reports suggest that insect-transmitted viruses severely impact Georgia-grown cucurbits [21,22,23,24,25,26,27]. Therefore, symptomatic leaf tissues were collected to further understand the insect-transmitted disease in cucurbits (pumpkin and winter squash). Broadly, four different types of symptoms, leaf (I) marginal yellowing (PK1), (II) cupping and shoestring (PK2), (III) mosaic and vein clearing (PK3), (IV) thickening, curling, crumpling, and vein clearing (PK4) in pumpkin (Figure 2A–D), and two different types of symptoms in winter squash (I) light and dark green mosaic and blistering (WS1) and (II) yellowing, thickening, and inward rolling (WS2) of leaves tissues (Figure 2E,F) were identified and were individually collected.

The application of HTS has emerged as the pivotal tool for detecting plant viruses in infected plant samples [32,33,34,35,36,37]. sRNA analysis of infected tissues showed the presence of five distinct viruses in the Georgia-grown pumpkin and winter squash leaves; three of them are whitefly-transmitted viruses (CuLCrV, CYSDV, and CCYV), and two of them are aphid-transmitted viruses (ZYMV and PRSV). HTS analysis suggests that the nucleotide sequence identity of ZYMV and PRSV Georgia isolates from pumpkin and winter squash were showing 80–100% with the reference genome sequence (PRSV; NC_001785) and (ZYMV; NC_003224), respectively (Figure 3).

The common occurrence of mixed infections involving two or more viruses, which may belong to the same or different families/genera, may result in intricate synergistic effects on the tripartite interactions between viruses, plants, and vectors and exacerbate the severity of their impact [38,39]. This study presents the prevalence and natural occurrence of ZYMV and PRSV in pumpkin and winter squash crops grown in Georgia. Consequently, a molecular and biological understanding of host–virus interaction, seasonal dynamics of viruses infecting cucurbit, and synergistic and antagonistic infection of ATV along with WTV is required for managing the viral diseases and might help stakeholders enhance profitability and sustainability.

Furthermore, qPCR was carried out to validate the presence of ZYMV and PRSV in symptomatic tissues. This study revealed a significant shift in viral prevalence between the two consecutive years (fall of 2022 and 2023). PRSV was more prevalent in pumpkin (56.25%) and winter squash (50.0%) in 2022, whereas ZYMV was abundant in 2023, infecting pumpkin (61.25%) and winter squash (42.50%). Interestingly, PRSV was detected in a negligible percentage (<1.0%) of samples using qPCR in these crops in 2023. The occurrence of mixed infections also declined substantially (Figure 4A,B). These findings highlight the dynamic nature of viral infections in cucurbit crops over time. A recent study in which surveys were conducted for three years (2016–2018) in different agricultural districts of Oklahoma, USA, suggests the temporal fluctuating distribution and prevalence of viruses in natural conditions. The presence of potyviruses like cucurbit aphid-born yellows virus (CABYV), PRSV-watermelon strain, watermelon mosaic virus (WMV), and ZYMV was confirmed using dot-immunobinding assay and qPCR. ZYMV prevalence had overtaken WMV over the three years [40]. Another study by Mondal et al. (2023) depicted a seasonal distribution of criniviruses (CCYV and CYSDV) infecting melon crops in the southern California and Arizona regions of the USA [41]. These studies further suggest differential accumulation of viral titer during mixed infection, which can lead to synergism or antagonism effects on the viruses. This finding suggests a possible competitive advantage over one and other viruses during mixed or co-infection. Our study found mixed infections of ZYMV and PRSV in addition to other WTVs, and their prevalence changes differentially during cucurbit production. The observed temporal shift in virus prevalence could be due to different factors that include environmental changes and vector dynamics. Environmental factors such as changes in weather patterns, and surrounding reservoirs (weed species) can alter the virus abundance. The temporal shift in vector species and vector genera density can result shift in virus prevalence across the years [42]. Therefore, a deeper investigation into these factors such as monitoring vector population dynamics, weather patterns, and virus–vector interactions could provide more insights into the underlying mechanisms.

To further confirm the ATV in pumpkin and winter squash, PCR and Sanger sequencing were performed. A phylogenetic study and heat map of ZYMV-encoded CP (Figure 5A,B) and HC-Pro (Figure 5C,D) suggested a close evolutionary history with the European isolates. ZYMV infects cucurbit crops primarily (winter squash, pumpkin, melon, sponge gourd, snake gourd, zucchini, and cucumbers) in several countries, including the USA [8,39,43,44]. In contrast, PRSV infected both cucurbits and papaya, suggesting a diverse host range [45]. PRSV-CP (Figure 5E,F) and PRSV-NIa-Vpg (Figure 5G,H) based phylogeny shows its closer relatedness with other PRSV isolates reported from Australia (KX655860, year 2014), Papua New Guinea (MH404261, year 2016), Spain (OR477277, year 2023), USA (OM687239, year 2019), and other countries. In addition, for potyviruses, phylogeny depends on the molecular and/or ecological mechanisms involved in adaptation to different plant species [46]. Moreover, human activities, especially trade and farming, have fostered and spread potyviruses and their aphid vectors worldwide [47,48].

To our knowledge, this is the first study suggesting single or mixed infections of ZYMV- and PRSV-infected pumpkin and winter squash in Georgia, USA. Single and mixed infections of ZYMV, PRSV-W, and WMV were also reported among the cucurbit crops, particularly with watermelon, collected from several states of the USA (Arizona, Florida, Mississippi, Oklahoma, and Texas) in 2010 and 2011 [17]. In mixed infection, tools of genetic variability such as frequent mutations, recombination, and reassortments in the viral genome assist in evolving viruses rapidly, altering the defense in the host and breaking down the resistance of cultivars [38]. However, the development and cultivation of resistant cultivars is the first and foremost component of integrated pest management strategies [49] and a sustainable approach to crop protection [50]. Understanding the incidence of mixed infections is crucial for developing resistant cultivars and stable management strategies against insect-transmitted viruses and diseases. In addition, the diagnosis of viruses in mixed infection is a critical aspect of the management of plant viruses due to the lack of readily available chemicals to control viruses. Lacking effective diagnosis and managing the prevalent ATV along with WTV hinder winter squash and pumpkin production in the region. Therefore, a more comprehensive study is needed to understand the possible mechanism of infection and pathogenesis of ATV together with WTV in cucurbit crops. The findings of this study will serve as an important foundation for future research aimed at understanding the complex interactions between aphid- and whitefly-transmitted viruses in cucurbit crops.

## Figures and Tables

**Table 1:** Primers used for the detection of viruses in pumpkin and winter squash leaf samples collected during the fall of 2022 and 2023 in Tifton, Georgia, USA.

Primer Name	Sequence (5′-3′)	Tm (°C)	Amplicon Size	References
qPCR primers				
ZYMV_qPCR_CP_F	CAGCGGAGGCATACATAGAAA	62	99	This study
ZYMV_qPCR_CP_R	CGTATCGAGCCAAACTCCTATC			
ZYMV_qPCR_HC-Pro_F	GGTACTCGTAAGTTGGCCATAG	62	100	This study
ZYMV_qPCR_HC-Pro_R	TGAGCGGCTTCTTCTCAATAC			
PRSV_qPCR_HC-Pro_F	CACATGACAGCCATCAACAAC	62	96	This study
PRSV_qPCR_HC-Pro_R	TCTCACGATCTCTCGAAGACTAT			
PRSV_qPCR_CP_F	GGAGATGGGAGAAGCCTATTTG	62	107	This study
PRSV_qPCR_CP_R	CCGTGTTGGGATTGCACTATAA			
Full-length primers				
ZYMV_CP_FL_F	TCAGGCACTCAGCCAACTG	63	837	This study
ZYMV_CP_FL_R	CTGCATTGTATTCACACCTAGG			
ZYMV_HC-Pro_FL_F	TCGTCGCAACCGGAAGTTC	63	1368	This study
ZYMV_HC-Pro_FL_R	GCCAACTCTGTAATGTTTCATCTC			
PRSV_CP_FL_F	TCTAAAAATGAGGCTGTGGATGC	65	861	This study
PRSV_CP_FL_R	GTTGCGCATACCCAGGAGAGA			
PRSV_Nia_FL_F	GGTTTCTCCGCACGACA	65	567	This study
PRSV_Nia_FL_R	TTCGTGATGAACTAATTTCGAG			

**Table 2:** PRSV and ZYMV were identified in sRNA read from pumpkin and winter squash grown during the fall of 2023 in Tifton, Georgia, USA.

Virus Detected	Crops	Sample ID	Genome Size of Refseq (nt)	Total sRNA Reads	^€^ Reads Matching to Virus	^¶^ Coverage (%)	^¥^ Nucleotide Identity (%)
ZYMV	Pumpkin	PK1	9591	21,632,823	99 (0.00045)	9591 (100)	100
		PK2		22,698,801	15,845 (0.069)	8550 (89.2)	89.15
		PK3		30,127,054	2,980,951 (9.89)	9006 (93.9)	93.90
		PK4		20,586,748	41,992 (0.20)	8866 (92.4)	92.44
	Winter squash	WS1		25,536,117	1,742,326 (6.822)	9001 (93.9)	93.85
		WS2		26,949,961	1,375,873 (5.10)	8917 (93)	92.97
PRSV	Pumpkin	PK1	10,326	21,632,823	83 (0.00038)	10,326 (100)	100
		PK2		22,698,801	483,543 (2.13)	9264 (89.7)	89.72
		PK3		30,127,054	324,041 (1.07)	9362 (90.7)	90.66
		PK4		20,586,748	109 (0.00052)	9694 (93.8)	93.88
	Winter squash	WS1		25,536,117	139,751 (0.54)	9304 (90.1)	90.10
		WS2		26,949,961	1341 (0.0049)	8558 (82.9)	82.88

^€^ Reads matching the virus, values in parentheses represent the percentage of total reads aligning with the virus genome. ^¶^ Coverage–percentage of the GenBank genome covered by contigs assembled from the sample. ^¥^ Nucleotide identity percentage, identity to the GenBank sequence with the highest nucleotide identity in BLAST of all contigs aligned to the sequence.

## Data Availability

All the sequence data generated were submitted to NCBI GenBank and the accession numbers are included in the text.

## Supplementary Materials

The following supporting information can be downloaded at https://www.mdpi.com/article/10.3390/v17020233/s1. Table S1: Cultivars and scientific names of pumpkin and winter squash cultivated in Tifton, Georgia, USA, during fall 2022 and 2023; Table S2: Crops, year, seed sowing date, row-to-row spacing, plant-to-plant spacing, and fertilizer rate applied in pumpkin and winter squash cultivated in Tifton, Georgia, USA, during the fall 2022 and 2023; Table S3: Common viruses in cucurbits other than ZYMV and PRSV with their reference sequences; Table S4: Sequences used for generating phylogenetic trees and heat maps.
