# Supplementary material for: Prevalence of Aphid-Transmitted Potyviruses in Pumpkin and Winter Squash in Georgia, USA
Source: Viruses. 2025 Feb 8;17(2):233. doi: 10.3390/v17020233 (PMC11860210; doi:10.3390/v17020233)
Supplement: Supplementary file 1 [file viruses-17-00233-s001.zip › viruses-3417454-supplementary.pdf]

Supplementary materials

Table S1. Cultivars and Scientific names of pumpkin and winter squash cultivated in Tifton, Georgia, USA, during Fall 2022 and 2023

Pumpkin		Winter squash	
Cultivars	Scientific name	Cultivars	Scientific name
Spartan	*Cucurbita pepo*	Tay Belle	*Cucurbita pepo*
Secretariat	*Cucurbita pepo*	Table Ace	*Cucurbita pepo*
Zeus[ϑ]	*Cucurbita maxima*	Sweet Mama	*Cucurbita maxima.*
Big Loretta	*Cucurbita maxima*	La Estrella	*Cucurbita moschata*
Orange Bulldog*[ȝ]	*Cucurbita maxima*	Golden Hubbard	*Cucurbita maxima*
		Waltham	*Cucurbita moschata*
		Ceres*	*Cucurbita moschata*
		Atlas	*Cucurbita moschata*
		Ultra HP*	*Cucurbita moschata*
		Genesis*	*Cucurbita moschata*

*Cultivars not cultivated in 2022 and added in 2023. [ϑ]Cultivar with intermediate resistance with zucchini yellow mosaic virus. [ȝ]Cultivar with tolerance to potyvirus. The pumpkin and winter squash seeds were procured from Seedway, LLC., NY, USA (https://www.seedway.com/), except Orange Bulldog which was sourced from University of Georgia (UGA), Athens, GA, USA.

Table S2. Crops, Year, Seed sowing date, row-to-row spacing, plant-to-plant spacing, and fertilizer rate applied in pumpkin and winter squash cultivated in Tifton, Georgia, USA, during Fall 2022 and 2023

Crops	Year	Seed sowing date	Row to row spacing (cm)	Plant to plant spacing (cm)	Fertilizer (kg/ha)
Pumpkin	2022	01 Aug	183	122	168 N; 134.4 P_2O_5; 112 K_2O
	2023	09 Aug	183	122	168 N; 134.4 P_2O_5; 112 K_2O
Winter squash	2022	01 Aug	183	91.5	168 N; 134.4 P_2O_5; 112 K_2O
	2023	09 Aug	183	91.5	168 N; 134.4 P_2O_5; 112 K_2O

Table S3. Common viruses in cucurbits other than ZYMV and PRSV with their reference sequences accession number aligned

Sl. No.	Viruses	Species (International Committee on Taxonomy of Viruses)	Genome	NCBI GeneBank Accession No.
1	Cucurbit chlorotic yellows virus (CCYV)	-	RNA 1	NC_018173
			RNA 2	NC_018174
2	Cucurbit yellow stunting disorder virus (CYSDV)	*Crinivirus cucurbitae*	RNA 1	NC_004809
			RNA 2	NC_004810
3	Cucurbit leaf crumple virus (CuLCrV)	*Begomovirus cucurbitae*	DNA A	NC_002984
			DNA B	NC_002985
4	Cucumber vein yellowing virus (CVYV)	*Ipomovirus cucumisvenaflavi*	RNA	NC_006941

5	Cucurbit aphid borne yellows virus (CABYV)	-	RNA	NC_026508
6	Melon severe mosaic virus (MeSMV)	-	RNA	NC_033832
7	Squash leaf curl virus (SLCV)	*Begomovirus cucurbitapeponis*	DNA A	NC_001936
			DNA B	NC_001937
8	Squash vein yellowing virus (SqVYV)	*Ipomovirus cucurbitavenaflavi*	RNA	NC_010521
9	Watermelon chlorotic stunt virus (WmCSV)	*Begomovirus citrulli*	DNA A	NC_003708
			DNA B	NC_003709
10	Watermelon crinkle leaf-associated virus 1 (WCLaV-1)	*Coguvirus citrulli*	RNA	NC_079048
11	Watermelon crinkle leaf-associated virus 2 (WCLaV-2)	*Coguvirus henanense*	RNA	NC_079050
12	Zucchini tigre mosaic virus (ZTMV)	*Potyvirus pepotigris*	RNA	NC_023175

Table S4. Sequences used for generating phylogenetic trees and heat maps.

Virus/Gene	NCBI GeneBank Acc. no.	Country	Host	Year
ZYMV/CP	PQ685676	USA	*Cucurbita maxima*	2023
ZYMV/CP	PQ685677	USA	*Cucurbita moschata*	2023
ZYMV/HC-pro	PQ685678	USA	*Cucurbita maxima*	2023
ZYMV/HC-pro	PQ685679	USA	*Cucurbita moschata*	2023
PRSV/CP	PQ685680	USA	*Cucurbita maxima*	2023
PRSV/CP	PQ685681	USA	*Cucurbita moschata*	2023
PRSV/Nia-Vpg	PQ685682	USA	*Cucurbita maxima*	2023
PRSV/Nia-Vpg	PQ685683	USA	*Cucurbita moschata*	2023
PRSV/CP and Nia-Vpg	OP947578	USA	*Citrullus lanatus*	2022
PRSV/CP and Nia-Vpg	OQ335837	Italy	*Cucurbita pepo*	2023
PRSV/CP and Nia-Vpg	MZ405666	Israel	*Cucurbita pepo*	2021
PRSV/CP and Nia-Vpg	X67673	Taiwan	*Cucumis metuliferus*	1994
PRSV/CP and Nia-Vpg	MT470188	USA	*Carica papaya*	1984
PRSV/CP and Nia-Vpg	MH974110	Ecuador	*Carica papaya*	2018
PRSV/CP and Nia-Vpg	PP256253	France	*Cucumis sativus*	2024
PRSV/CP and Nia-Vpg	KY271954	USA	*Carica papaya*	2014
PRSV/CP	MN203186	Mexico	*Carica papaya*	2014
PRSV/CP and Nia-Vpg	MN203187	Mexico	*Carica papaya*	2014
PRSV/CP and Nia-Vpg	KX655860	Australia	*Cucurbitaceae*	2014
PRSV/CP and Nia-Vpg	MH404261	Papua New Guinea	*Carica papaya*	2016
PRSV/CP and Nia-Vpg	OM687239	USA	*Cucumis melo*	2019
PRSV/CP and Nia-Vpg	OR477277	Spain	*Carica papaya*	2023
PRSV/CP and Nia-Vpg	KT275937	Columbia	*Carica papaya*	2014

PRSV/CP and Nia-Vpg	PP035971	Phillipines	*Carica papaya*	2023
PRSV/CP and Nia-Vpg	OK633883	Vietnam	*Carica papaya*	2015
PRSV/CP and Nia-Vpg	MT470190	Thailand	*Carica papaya*	2013
PRSV/CP	PP503024	East Timor	*Cucurbitaceae*	2011
PRSV/CP and Nia-Vpg	MG030689	Brazil	*Cucumis melo*	2015
PRSV/CP and Nia-Vpg	KP462721	Brazil	*Fevillea cordifolia*	2012
PRSV/CP and Nia-Vpg	DQ374152	Brazil	*Cucurbita pepo*	2006
PRSV/Nia-Vpg	OL677454	India	*Carica papaya*	2021
PRSV/Nia-Vpg	MT470190	Pakistan	*Carica papaya*	2015
PRSV/Nia-Vpg	MH397222	Bangladesh	*Carica papaya*	2016
PRSV/Nia-Vpg	KX655874	Australia	*Cucurbita spp*	2014
PRSV/Nia-Vpg	PP503024	Taiwan	*Cucurbita pepo*	2024
PRSV/Nia-Vpg	OK465456	China	*Cucumis melo*	2017
ZYMV/CP/HC-Pro	KF976712	Czech Republic	*Cucurbita pepo*	2013
ZYMV/CP and HC-Pro	DQ124239	Slovakia	*Cucurbita pepo*	2005
ZYMV/CP and HC-Pro	KU244513	Greece	*Citrullus lanatus*	2014
ZYMV/CP and HC-Pro	ON604841	Hungary	*Cucurbita pepo*	2022
ZYMV/CP and HC-Pro	PP256252	Brazil	*Cucurbita pepo*	2024
ZYMV/CP and HC-Pro	OR233209	Israel	*Cucumis sativus*	2023
ZYMV/CP and HC-Pro	OR879104	Spain	*Cucumis melo*	2023
ZYMV/CP and HC-Pro	OP357945	Sudan	*Cucumis sativus*	2022
ZYMV/CP and HC-Pro	OP947584	Cote d Ivoire	*Cucumis sativus*	2022
ZYMV/CP and HC-Pro	MT497463	Kenya	*Cucurbita moschata*	2018
ZYMV/CP	GU586790	Brazil	*Trichosanthes cucumerina*	2009
ZYMV/CP and HC-Pro	MF072712	Trinidad and Tobago	*Cucurbita moschata*	2015
ZYMV/CP and HC-Pro	OR233210	Italy	*Cucurbita pepo*	2023
ZYMV/CP and HC-Pro	KT598222	Argentina	*Cucurbita maxima*	2011
ZYMV/CP and HC-Pro	KX499498	Spain	*Cucurbita pepo*	2016
ZYMV/CP and HC-Pro	MH042024	South Korea	*Cucurbita pepo*	2016
ZYMV/CP and HC-Pro	OM471983	UK	*Cucurbita pepo*	2022
ZYMV/CP and HC-Pro	JN192405	USA	*Cucurbita pepo*	2007
ZYMV/CP and HC-Pro	OQ847410	France	*Cucumis melo*	2023
ZYMV/CP and HC-Pro	MK124612	USA	*Cucurbita moschata*	2016

ZYMV/CP	OK376504	USA	*Cucurbita pepo*	2016
ZYMV/CP and HC-Pro	MN598567	Australia	*Cucurbita pepo*	2016
ZYMV/CP and HC-Pro	OP947577	Singapore	*Cucumis sativus*	2022
ZYMV/CP and HC-Pro	AJ515911	China	*Citrullus lanatus*	2002
ZYMV/CP	EF178505	Poland	*Cucurbita pepo*	2006
ZYMV/CP	AF12792	Taiwan	*Luffa cylindrica*	1999
ZYMV/CP and HC-Pro	Z73124	Peru	*Nicotiana benthamiana*	1996
ZYMV/HC-Pro	KT778297	India	*Cucumis anguria*	2012
ZYMV /HC-Pro	ON604832	Germany	*Cucurbita pepo*	2022
ZYMV/HC-Pro	LC799407	Egypt	*Cucurbita pepo*	2022
